# A review of hyperspectral image analysis techniques for plant disease detection and identif ication

**DOI:** 10.18699/VJGB-22-25

**Published:** 2022-03

**Authors:** A.F. Cheshkova

**Affiliations:** Siberian Federal Scientif ic Center of AgroBioTechnology of the Russian Academy of Sciences, Krasnoobsk, Novosibirsk region, Russia

**Keywords:** hyperspectral technologies, plant diseases, image analysis, spectral analysis, гиперспектральные технологии, болезни растений, анализ изображений, спектральный анализ

## Abstract

Plant diseases cause signif icant economic losses in agriculture around the world. Early detection, quantif ication and identif ication of plant diseases are crucial for targeted application of plant protection measures in crop production. Recently, intensive research has been conducted to develop innovative methods for diagnosing plant diseases based on hyperspectral technologies. The analysis of the ref lection spectrum of plant tissue makes it possible to classify healthy and diseased plants, assess the severity of the disease, differentiate the types of pathogens, and identify the symptoms of biotic stresses at early stages, including during the incubation period, when the symptoms are not visible to the human eye. This review describes the basic principles of hyperspectral measurements and different types of available hyperspectral sensors. Possible applications of hyperspectral sensors and platforms on different scales for diseases diagnosis are discussed and evaluated. Hyperspectral analysis is a new subject that combines optical spectroscopy and image analysis methods, which make it possible to simultaneously evaluate both physiological and morphological parameters. The review describes the main steps of the hyperspectral data analysis process: image acquisition and prepro cessing; data extraction and processing; modeling and analysis of data. The algorithms and methods applied at each step are mainly summarized. Further, the main areas of application of hyperspectral sensors in the diagnosis of plant diseases are considered, such as detection, differentiation and identif ication of diseases, estimation of disease severity, phenotyping of disease resistance of genotypes. A comprehensive review of scientif ic publications on the diagnosis of plant diseases highlights the benef its of hyperspectral technologies in investigating interactions between plants and pathogens at various measurement scales. Despite the encouraging progress made over the past few decades in monitoring plant diseases based on hyperspectral technologies, some technical problems that make these methods diff icult to apply in practice remain unresolved. The review is concluded with an overview of problems and prospects of using new technologies in agricultural production

## Abstract (ru)

Болезни растений приводят к значительным экономическим потерям в секторе сельскохозяйственного производства во всем мире. Раннее выявление, количественная оценка и идентификация болезней имеют решающее значение для целенаправленного применения мер защиты в растениеводстве. В настоящее время ведутся интенсивные научные исследования по разработке инновационных методов диагностики болезней растений, основанных на гиперспектральных технологиях. Анализ спектра отражения растительной ткани позволяет проводить классификацию здоровых и больных растений, оценивать тяжесть заболевания, дифференцировать виды патогенов и выявлять симптомы биотических стрессов на ранних стадиях, в том числе в инкубационный период, когда симптомы не видны человеческому глазу. В обзоре описаны основные принципы измерения спектра отражения растительной ткани. Обсуждаются и оцениваются возможности применения различных типов гиперспектральных сенсоров и платформ для диагностики болезней растений. Гиперспектральный анализ является новой областью, соединяющей в себе методы оптической спектроскопии и методы анализа изображений, которые позволяют одновременно оценивать как физиологические, так и морфологические параметры. Описаны главные этапы анализа гиперспектральных данных: получение и предварительная обработка изображения; извлечение и обработка данных; моделирование и анализ данных. Приведен перечень алгоритмов и методов, применяемых на каждом из этапов. Рассмотрены основные области применения гиперспектральных сенсоров в диагностике болезней растений, такие как обнаружение болезни, дифференциация и идентификация типа заболевания, оценка степени поражения, оценка устойчивости генотипов. Приведен всесторонний обзор научных публикаций, подчеркивающий преимущества гиперспектральных технологий при исследовании взаимодействий между растениями и патогенами в различных масштабах измерений. Несмотря на обнадеживающий прогресс, достигнутый за последние несколько десятилетий в мониторинге болезней растений на основе гиперспектральных технологий, остаются нерешенными некоторые технические проблемы, препятствующие применению этих методов на практике. В заключение обсуждаются проблемы и перспективы практического использования новых технологий в сельскохозяйственном производстве

## Introduction

Plant diseases cause crop losses, reduce the quality of agricultural products and can even threaten human health. Farmers need modern and effective tools for early detection and identification of plant diseases (Mahlein et al., 2019b). Traditional diagnostic methods such as visual assessment and microbiological laboratory analysis are time-consuming and labor-intensive, which limits their application in largescale farms.

Currently, new non-invasive methods for diagnosing plant diseases using sensor technologies, robotics, computer vision and machine learning are rapidly developing (Singh A. et al., 2015; Demidchik et al., 2020; Zheng et al., 2021). These methods are high throughput and provide a real-time support for assessing a range of physiological parameters (Walter et al., 2015). A large amount of information obtained from modern sensors is transformed into new knowledge using computer data processing and modeling, reducing the distance from fundamental science to practical implementation (Afonnikov et al., 2016; Tardieu et al., 2017). New approaches allow, due to automation, to significantly speed up the diagnosis of diseases and increase its accuracy by eliminating the human subjectivity (Fahlgren et al., 2015; Lobos et al., 2017).

At present, a variety of imaging methods are being used for plant diseases detection, such as fluorescence imaging, thermal infrared imaging, visible RGB imaging, imaging spectroscopy and other techniques (Bock et al., 2010; Li L. et al., 2014).

**Fig. 1.**
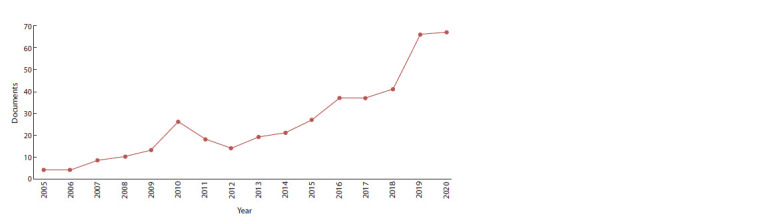
Number of published articles by year on plant diseases with hyperspectral data (Scopus).

Among them, hyperspectral imaging technique comes with numerous advantages (Mahlein, 2016; Mahlein et al., 2018; Dubrovskaya et al., 2018). According to the Scopus statistics, there are 412 relevant papers from 2005 to 2020 where ‘plant disease’ and ‘hyperspectral’ are used as key words for the search (Fig. 1). Hyperspectral analysis combines optical spectroscopy and image analysis methods, allowing both physiological and morphological parameters to be evaluated simultaneously.

The aim of the paper is to provide the reader with an overview of modern technologies for the diagnosis of plant diseases based on the analysis of hyperspectral images. The first part of the article discusses the main principles and tools of hyperspectral technologies. Next, algorithms and methods for analyzing hyperspectral images are described. Further, the main areas of application of hyperspectral sensors in the diagnosis of plant diseases are considered. The paper is concluded with some problems and prospects of using new technologies.

## Basic principles and tools of hyperspectral technologies

Interaction of light (electromagnetic radiation) and plants

Light can interact with plant tissue in the following ways: reflection, scattering, absorption and transmission. The reflectance characteristic of a plant results from the biochemical compounds present in the leaves, and the physical characteristics of leaves (Mishra et al., 2017). The interaction between light and plants also depends on the wavelength. In the visible wavelength range (400–700 nm), the surface of the plant has a low reflectivity due to the absorption of light by photosynthetic pigments (chlorophylls, anthocyanins and carotenoids). In the near infrared (700–1100 nm), the reflectance increases due to light scattering in the intercellular space. In the short wave infrared range (1100–2500 nm), healthy plants have a low reflectance due to the absorption of light by water, proteins and other carbon components (Lowe et al., 2017). The green color of the leaf is consistent with the characteristic reflection peak at 550 nm.

Spectral profiles of healthy and diseased plants can differ. As a result of the impact of biotic and abiotic stressors, the biochemical composition of plant tissues changes, which is reflected in the change in the color and shape of leaves, transpiration rate, canopy morphology, and, consequently, in the spectral characteristics of plants (Zhang J. et al., 2019). Moreover, each individual interaction of a plant and a pathogen has certain spatial and temporal dynamics, and these processes affect different ranges of the electromagnetic spectrum. For example, a change in photosynthetic activity caused by pathogens leads to a change in reflectivity in the visible range of the spectrum. Changes at the cellular level have a large impact on the near infrared spectrum. Tissue necrosis leads to increased reflection in the shortwave infrared range (Zhang N. et al., 2020).

Such relationships between cause and consequence can be used to study the biochemistry of plants and to perform controlled experiments

Hyperspectral sensors and platforms

The basic principle of hyperspectral sensors is comparable to the principle behind RGB and multispectral cameras (Thomas et al., 2018b). All these systems measure the amount of light reaching the sensor and store the information. Unlike RGB cameras (3 spectral bands) or multispectral cameras (< 20 spectral bands), a hyperspectral sensor measures up to several hundred bands of the electromagnetic spectrum in the wavelength range of the sensor. Each of these spectral bands measures only a few nanometers of the electromagnetic spectrum, leading to a high spectral resolution of the hyperspectral sensor.

There are two main types of sensors: image sensors and non-imaging sensors. Non-imaging sensors measure the average reflectance spectrum in a certain area of a surface without storing spatial information. The size of the averaging area depends on the focal length, angle of view and distance to the object. Most non-imaging sensors are portable and do not require complicated measurement platforms. They have a wide spectral range (300–2500 nm), a high spectral resolution (1–3 nm), and low weight (1–5 kg). The most popular among them are spectrometers ASD FieldSpec (Analytical Spectral Devices Inc., USA), SVC (Spectral Vista Corporation, USA), ImSpector (Spectral Imaging Ltd., Finland).

These devices are widely used in laboratory, greenhouse and field conditions (Naidu et al., 2009; Zhang J. et al., 2017; Couture et al., 2018; Bohnenkamp et al., 2019; Mahlein et al., 2019a). There are also micro-spectrometers such as the STS-VIS spectrometer (Ocean Optics Inc., USA) suitable for use with UAVs (Burkart et al., 2015). Since early symptoms of plant disease often appear below 1 mm, their detection with spectrometers is limited. This is due to the averaging of the spectrum of healthy and diseased tissue in the measurement area (Mahlein et al., 2012).

**Fig. 2.**
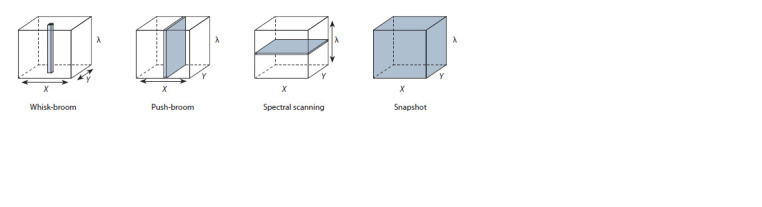
Acquisition approaches of hyperspectral images. Scanning directions are shown by arrows, and gray areas show data acquired each time.

Hyperspectral image sensors form a spectral profile for each individual pixel, thereby combining spectral and spatial resolution. The resulting image is a three-dimensional data array (hypercube) containing two dimensions of spatial information and additionally one dimension of spectral information. Depending on the type of sensors used, there are four ways to obtain a hypercube of data (Fig. 2): whiskbroom, push-broom, spectral scanning, and snapshot (Wu, Sun, 2013).

Hyperspectral image sensors usually cover a limited spectral range: VNIR (300–1000 nm) or SWIR (1000–2500 nm) with a spectral resolution of 1–7 nm. Spatial resolution ranges from micrometers to centimeters depending on the distance to the object and sensor characteristics

In the case of using point or line scanning sensors (whiskbroom, push-broom), it is necessary to move the object or the camera to register the spectrum of each individual point or line. In scientific research, the most commonly used scanning cameras are Specim (Spectral Imaging Ltd., Finland), Headwall (Headwall Hyperspec Ltd., Canada), Photonfocus (Photonfocus AG, Switzerland), Pika L (Resonon Inc., USA). Most hyperspectral scanning cameras in the laboratory are installed on specialized mobile platforms that provide linear movement and stabilization of the camera (Leucker et al., 2016; Yeh et al., 2016). Stationary rail systems are used in greenhouses (Thomas et al., 2018a). Vehicles (Vigneau et al., 2011; Williams et al., 2017) or UAVs (Huang W. et al., 2007; Abdulridha et al., 2019) are used in the field. The disadvantage of scanning sensors is the relatively long image acquisition time, depending on the size of the measured area, which complicates the shooting of moving objects. This disadvantage is eliminated in portable Specim IQ camera with a built-in scanner (Behmann et al., 2018; Alt et al., 2020; Barreto et al., 2020).

Spectral scanning sensors use LCTF filters that pass only certain wavelengths changing rapidly during shooting (Choudhary et al., 2009; Wang et al., 2012). These sensors create 2D spatial images for each wavelength in the spectral range. Their use does not require moving the object or camera to obtain a hypercube. The acquisition time is mainly dependent on the exposure time, which is generally faster than point or line scans. If the object is moving, then this measuring principle can lead to inconsistent spectra, since the individual bands are observed at different times.

Recently, snapshot sensors that do not require scanning an object to obtain a hypercube have been developed. They use the mosaic principle of conventional RGB cameras. These sensors provide a significantly higher image recording rate, but lower spatial resolution compared to traditional ones. Well-known cameras of this type are Rikola, Senop (Senop Ltd., Finland), Ultris, FireFleye (Cubert Ltd., Canada). The compact size, short image acquisition time and the ability to create a sequence of hyperspectral images of a moving object make them optimal for use in UAVs (Aasen et al., 2015; Sankaran et al., 2015; Franceschini et al., 2019).

## Hyperspectral image processing methods and algorithms

From the data analysis perspective the use of multi-scale datasets of hyperspectral images, characterized by a huge amount of data with a high level of collinearity, is a very challenging, emerging topic that requires non-trivial solutions. To face this challenge, the methods of discriminant and cluster analysis, machine learning, and neural networks have been successfully adopted (ElMasry et al., 2016; Lowe et al., 2017).

Available software tools for hyperspectral image analysis process are ENVI (Research Systems Inc.), MATLAB (The Math-Works Inc.), Python (Python Software Foundation), R (R Software Foundation).

**Fig. 3.**
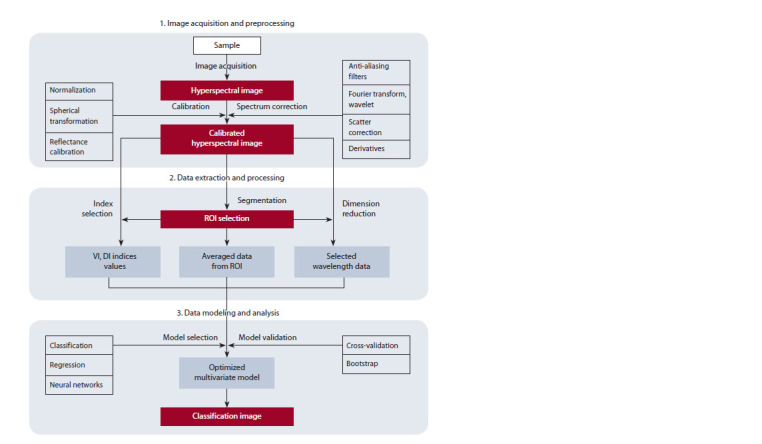
Flowchart of a series of typical steps for analyzing hyperspectral image data.

The hyperspectral image analysis process usually includes the following steps (Fig. 3): (1) image acquisition and preprocessing, (2) data extraction and processing, (3) data modeling and analysis.

Image acquisition and preprocessing

The first important step in the analysis of plant diseases is to obtain high-quality hyperspectral images that meet the objectives of research. The right choice of sensors and platforms, the correct setting of the spatial and spectral resolution, lighting scheme, scan rate, frame rate and exposure time are prerequisites for obtaining accurate results (Wu, Sun, 2013).

The next step is image preprocessing, which includes calibration and spectrum correction. The goals of the calibration process are to standardize the spectral and spatial axes of the hyperspectral image, evaluate accuracy and reproducibility of the acquired data under different operating conditions, eliminate curvature effect and instrumental errors (Rinnan et al., 2009; Vidal, Amigo, 2012).

The standard practice is reflection calibration, which uses two reference images, black and white. The black image is acquired when the camera lens is completely covered with its opaque cap. The white reference image is obtained using a white surface board (e. g. Teflon) with a reflectivity of about 99.9 % to obtain the highest possible intensity for each pixel at each wavelength. These two reference images are then used to correct the raw hyperspectral images by using the following equation:

**Formula. 1.**
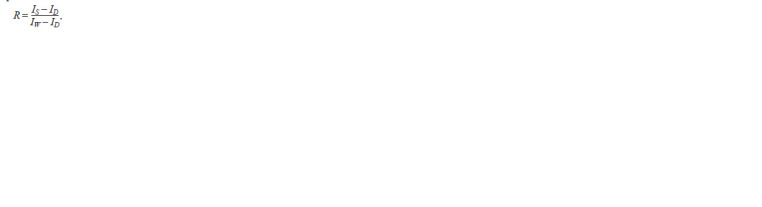
Formula

where R is the corrected hyperspectral image, IS is the raw hyperspectral image, ID is the dark image, and IW is the white reference image.

To eliminate the effect of surface curvature, spectral image normalization (Polder et al., 2004), adaptive spherical transform (Tao, Wen, 1999) or Lambert transform (Gomez- Sanchis et al., 2008) are used during calibration.

The goal of spectrum correction is to improve image quality (Savitzky, Golay, 1964; Barnes et al., 1989; Burger, 2006; Esquerre et al., 2012). For example, smoothing algorithms (moving average, Savitzky–Golay, median filter, and Gaussian filter), as well as Fourier and wavelet transforms, are used to reduce noise from the spectral data. The first and second derivatives are used to correct the shift of the spectrum baseline. Multiplicative scattering correction (MSC) and standard normal variate (SNV) are used to reduce the spectral variability due to scattering.

Data extraction and processing

At this step of hyperspectral image analysis process, image segmentation is performed and features are selected for further analysis.

Image segmentation is used as a pre-processing step and is typically performed before the formal spectral analysis in order to extract the target objects from the background or form a mask for the formation of the region of inte rests (ROIs) for further information extraction. The following segmentation methods are used: threshold-based (Pandey et al., 2017); K- means (Behmann et al., 2014); watershed algorithm (Li J. et al., 2019); edge detection (Sun et al., 2017; Williams et al., 2017).

Feature extraction can be considered to be the most important step in hyperspectral-based classification. Its goal is to extract and form new feature vectors for plant disease detection by combining and optimizing the spectral, spatial and texture features, then feed them to a set of classifiers or machine learning algorithms.

Vegetation indices (VI) or disease indices (DI) can be used as features (Huete et al., 2002; Gitelson et al., 2006; Mahlein et al., 2013; Candiago et al., 2015). In this case, only a small number of wavelengths are required for analysis. When analyzing the entire spectrum, the following methods are used to reduce the dimension and eliminate autocorrelations: principal component analysis; minimum noise fraction algorithm; linear discriminant analysis; stepwise discriminant analysis; partial least square discriminant analysis (Steddom et al., 2003; Delalieux et al., 2007; Naidu et al., 2009; Moshou et al., 2011; Yuan et al., 2014b; Zhou et al., 2019).

Data modeling and analysis

The last step in image analysis is to select a model and apply it to the data. Depending on the objectives of the study, these can be classification models (for diagnosing and differentiating diseases), or regression models (for predicting and assessing the relationship between the target variables and the spectral response).

The most commonly used models are: • classification models of machine learning and neural networks: spectral angle mapper, support vector machine, k-nearest neighbor, maximum likelihood (Moshou et al., 2004; Liu et al., 2010; Rumpf et al., 2010; Yeh et al., 2013; Li Y. et al., 2017); • regression models: multiple linear regression, binary logistic regression, partial least squares regression, Dirichlet aggregation regression (Huang W. et al., 2007; Singh D. et al., 2007; Yang et al., 2007; Huang J. et al., 2012).

Areas of application of hyperspectral technologies in diagnostics of plant diseases

The main tasks in the diagnosis of plant diseases are detection, differentiation, identification, assessment of the disease severity, assessment of the genotypes disease resistance. These tasks are solved at various levels of organization of living systems in the corresponding measurement scales.

Measurements at the cellular or tissue scales are carried out in laboratories using hyperspectral microscopes to observe fungal spores and detect metabolic changes in tissues caused by plant-pathogen interactions. Experiments at the cellular level are usually carried out in the context of fundamental research and to some extent for the identification of pathogens and the assessment of genotype resistance.

Measurements at the level of individual organs (leaf, ear, stem, root, fruit) and at the level of the whole plant are carried out in laboratory, greenhouse or field conditions with the aim of early detection and differentiation of the disease

Canopy-level measurements are more often applied in plant disease mapping and severity assessment.

Below is a brief overview of scientific publications on hyperspectral technologies in plant diseases diagnostics in the co

**Table 1.**
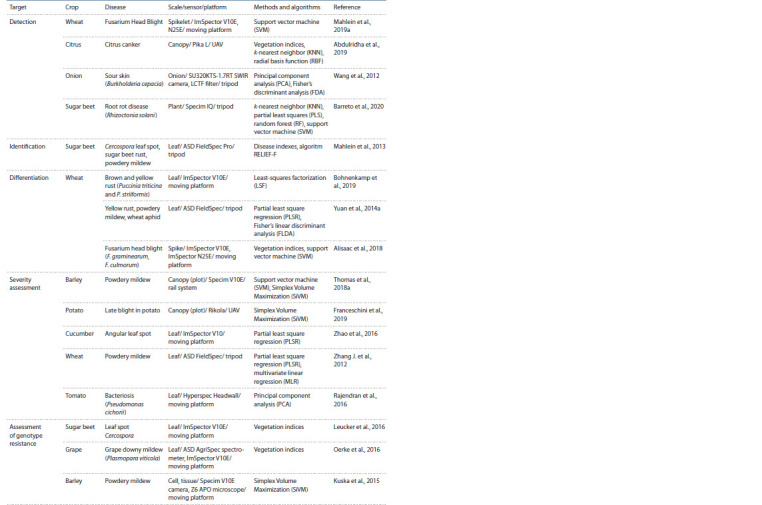
List of major contributions to different areas of application of hyperspectral images to plant diseases diagnostics

ntext of different areas of application (see the Table).

Disease detection

The aim of disease detection is to differentiate healthy and infected plants. In this case, the subject of research is only one specific disease, its symptoms and dynamics. A study of Mahlein et al., 2019a compares the feasibility of different sensors to characterize Fusarium head blight. Under controlled conditions, time-series measurements were performed with infrared thermography, chlorophyll fluorescence imaging, and hyperspectral imaging. Infrared thermography allowed the visualization of temperature differences within the infected spikelets beginning 5 days after inoculation. Also, on the 5th day, a disorder of the photosynthetic activity was confirmed by chlorophyll fluorescence imaging of spikelets. Pigment-specific simple ratio derived from hyperspectral imaging allowed discrimination between Fusarium-infected and non-inoculated spikelets on the 3rd day. Support vector machine method was used for classification. The classification accuracy was 78, 56 and 78 %, respectively.

A study of Abdulridha et al., 2019 compares two methods for detecting citrus canker with hyperspectral imaging. In the laboratory, a hyperspectral (400–1000 nm) imaging system was utilized for the detection of citrus canker at several disease development stages (i. e., asymptomatic, early, and late symptoms) by using two classification methods: (i) radial basis function (RBF) and (ii) k-nearest neighbor (KNN). The same imaging system mounted on a UAV was used to detect citrus canker on tree canopies in the orchard. The overall classification accuracy of the RBF was higher (94, 96, and 100 %) than the KNN method (94, 95, and 96 %) for detecting canker in leaves. Among the 31 studied vegetation indices, the water index (WI) and the Modified Chlorophyll Absorption in Reflectance Index (ARI and TCARI 1) more accurately detected canker in laboratory and in orchard conditions, respectively. The UAV-based technique achieved 100 % classification accuracy for identifying healthy and canker-infected trees.

Diseases identif ication and differentiation

In disease identification, the goal is to determine the type of pathogen affecting the plant. The subject of research is several types of diseases, their distinctive features.

Mahlein et al., 2013 developed specific spectral disease indices (SDIs) for the differentiation of diseases in crops. Sugar beet plants and three leaf diseases Cercospora leaf spot, sugar beet rust and powdery mildew were used as model system. Hyperspectral signatures of healthy and diseased sugar beet leaves were assessed with a nonimaging spectroradiometer at different development stages and disease severities of pathogens. Significant and most relevant wavelengths and two band normalized differences from 450 to 950 nm, describing the impact of a disease on sugar beet leaves, were extracted from the data-set using the RELIEF-F algorithm. To develop hyperspectral indices, the best weighted combination of a single wavelength and a normalized wavelength difference was searched. Healthy sugar beet leaves and leaves, infected with Cercospora leaf spot, sugar beet rust and powdery mildew were classified with a high accuracy and sensitivity (balanced classification accuracy: 89, 92, 87, and 85 %, respectively).

A study of Bohnenkamp et al., 2019 establishes a method for detecting and distinguishing between brown rust (Puccinia triticina) and yellow rust (P. striiformis) on wheat leaves based on hyperspectral imaging. The experiment was conducted at the leaf scale under controlled laboratory conditions. A reference spectrum from sporescale observations was used. Least-squares factorization was applied on hyperspectral images to unveil the presence of the spectral signal of rust spores in mixed spectra on wheat leaves. For the first time, this study shows an interpretable decomposition of the spectral reflectance mixture during patho-genesis.

Disease severity assessment

Quantitative diagnosis of plant disease severity is one of the main directions of hyperspectral disease analysis. The evaluation criteria for plant disease severity are often the disease index and incidence. In addition, according to the pathogens and symptoms they caused, the pigment content, water content, and even structural parameters are often regarded as indirect evaluation criteria

Zhao Y.-R. et al., 2016 used hyperspectral imaging to determine the spatial distribution of chlorophyll and carotenoid contents in cucumber leaves infected with angular spot. The pigment content was measured by biochemical analyzes. Partial least square regression (PLSR) models were used to develop quantitative analysis of the relationship between the disease severity, the spectra and the pigment contents. In addition, regression coefficients in PLSR models were employed to select important wavelengths for modeling. Finally, chlorophyll and carotenoid distributions in cucumber leaves with the angular spot infection were mapped by applying the optimal models pixel-wise to the hyperspectral images.

Zhang J. et al., 2012 detected wheat powdery mildew disease severity via spectral measurement and analysis. In this study, hyperspectral reflectances of normal and powdery mildew infected leaves were measured with a spectroradiometer in a laboratory. The severity of the disease was determined on a nine-point scale of the disease index. A total of 32 spectral features were extracted from the lab spectra and examined through a correlation analysis and an independent t-test associated with the disease severity. Two regression models: multivariate linear regression (MLR) and partial least square regression (PLSR) were developed for estimating the disease severity of powdery mildew. Based on the cross-validation result, seven spectral indices minimizing the relative root mean square error were selected. The PLSR model outperformed the MLR model, with a relative root mean square error of 0.23 and a coefficient of determination of 0.80 when using seven indices

Assessment of genotypes resistance

Analysis of the pathogen-host interaction makes it possible to determine the resistance of genotypes to a specific disease and is an important part of breeding. In breeding practice, phenotyping of plant genotypes is carried out by means of labor-intensive and expensive visual assessment. In this context, hyperspectral analysis is a promising noninvasive method for speeding up and automating traditional phenotyping methods

Leucker et al., 2016 evaluated the resistance of 5 different sugar beet genotypes to Cercospora leaf spot in their study. The experiment was carried out under controlled laboratory conditions. Lesions of Cercospora leaf spot were rated by classical quantitative and qualitative methods in combination with non-invasive hyperspectral imaging. It was found that the spectral characteristics of the affected leaf areas depend on the density of pathogen spores on the surface and on their spatial distribution. Accordingly, the number of conidia per diseased leaf area on resistant plant was lower. The assessment of lesion phenotypes by hyperspectral imaging with regard to sporulation may be an appropriate method for identifying subtle differences of genotypes in disease resistance.

Kuska et al., 2015 used a hyperspectral microscope to determine the resistance of barley cultivars to powdery mildew (Blumeria graminis). The reflection of inoculated and non-inoculated leaves was recorded daily with a hyperspectral linescanner in the visual (400–700 nm) and near infrared (700–1000 nm) range 3 to 14 days after inoculation. The susceptible genotypes showed an increase in reflectance in the visible range according to symptom development. However, the spectral signature of the resistant genotype did not show significant changes over the experimental period.

Problems and prospects of using hyperspectral technologies for the diagnosis of plant diseases

Despite the encouraging progress in monitoring plant diseases based on hyperspectral technologies made over the past few decades, some technical problems remain unresolved that make these methods difficult to apply in practice. Studies seeking solutions to these challenges will shape future trends

Currently, low-altitude, airborne and satellite multispectral systems are widely used in agricultural production to monitor the canopy based on vegetation indices (Hatfield, Pinter, 1993; Huang Y.B. et al., 2013). But reliable remote sensing monitoring of plant diseases and pests is usually achieved when symptoms are fully exhibited, which may be too late for guiding the prevention. Despite significant results in scientific research on the use of hyperspectral sensors for early detection of plant diseases, their practical application in field and greenhouse conditions in precision farming systems is still an unresolved problem.

Most of these studies have been conducted in controlled conditions, often utilizing artificial illumination and precisely regulating the directions of incoming light and reflected light being registered by positioning the camera or sensor at a defined angle toward the leaf tissue. The illumination conditions in the field are very different from laboratory ones, which creates enormous difficulties for reliably quantifying diseases in a natural canopy. Canopy regions located in sunlight appear much brighter than canopy layers situated in the shade. Tissue color depends on the angle of the tissue toward both the incoming sunlight and the reflected outgoing light. Heterogeneities in image brightness change from minute to minute. Therefore, setting a threshold for distinguishing between healthy and diseased tissue would mean taking the overall brightness of the specific image within the location into account, as well as the angle of incidence of light, which is currently a matter of intense research (Guo et al., 2013; Yu et al., 2017).

Another unsolved problem is to accurately detect a specific disease under realistic field conditions where several crop stressors may occur simultaneously. Currently, most monitoring studies or applications are conducted in experimental fields or areas with prior information about the type of pathogen. For an area that lacks corresponding information, it is challenging to achieve a reliable and accurate monitoring result. Many pathogens, as well as abiotic stressors, have similar symptoms and, therefore, a similar spectral signature. Some state-of-the-art algorithms, such as deep learning algorithms, may play an important role in differentiating biotic and abiotic stressors in field and greenhouse conditions (Liu et al., 2010; Mahlein et al., 2019b). Besides, it is necessary to promote the establishment of a knowledge base with the background information about diseases (i. e., geographical distribution, favorable habitats, soil types, climate conditions). The prior information may lower uncertainty in the monitoring of plant diseases

## Conclusion

Plant diseases are causing significant economic losses in the agricultural production around the world, especially given the climate change that has taken place in recent years. A promising technology for a non-invasive, fast, efficient and reliable way to detect and identify plant diseases is the use of hyperspectral sensors and platforms

New technologies are expanding human perception by providing information beyond the visible spectrum. The analysis of the reflection spectrum of plant tissue makes it possible to classify healthy and diseased plants, assess the severity of the disease, differentiate the types of pathogens, and identify the symptoms of biotic stresses at early stages, including during the incubation period, when the symptoms are not visible to the human eye

Due to the huge amount of information, the most promising methods for processing hyperspectral data are machine learning and neural networks. Currently, hyperspectral methods for diagnosing plant diseases are still at an early stage of development. In addition to its being an expensive technology, many technical difficulties limit its application in production. However, with advances in sensor technology and data analysis techniques, hyperspectral imaging can be expected to become one of the important tools for studying plant diseases.

## Conflict of interest

The authors declare no conflict of interest.
